# Misfolded proinsulin impairs processing of precursor of insulin receptor and insulin signaling in β cells

**DOI:** 10.1096/fj.201900442R

**Published:** 2019-08-01

**Authors:** Shiqun Liu, Xin Li, Jing Yang, Ruimin Zhu, Zhenqian Fan, Xiaoxi Xu, Wenli Feng, Jingqiu Cui, Jinhong Sun, Ming Liu

**Affiliations:** * Department of Endocrinology and Metabolism, Tianjin Medical University General Hospital, Tianjin, China;; † Department of Health Management, Tianjin Medical University General Hospital, Tianjin, China

**Keywords:** MIDY, proinsulin misfolding, insulin resistance, pancreatic, β cells, ER stress

## Abstract

Insulin resistance in classic insulin-responsive tissues is a hallmark of type 2 diabetes (T2D). However, the pathologic significance of β-cell insulin resistance and the underlying mechanisms contributing to defective insulin signaling in β cells remain largely unknown. Emerging evidence indicates that proinsulin misfolding is not only the molecular basis of mutant *INS*-gene–induced diabetes of youth (MIDY) but also an important contributor in the development and progression of T2D. However, the molecular basis of β-cell failure caused by misfolded proinsulin is still incompletely understood. Herein, using Akita mice expressing diabetes-causing mutant proinsulin, we found that misfolded proinsulin abnormally interacted with the precursor of insulin receptor (ProIR) in the endoplasmic reticulum (ER), impaired ProIR maturation to insulin receptor (IR), and decreased insulin signaling in β cells. Importantly, using db/db insulin-resistant mice, we found that oversynthesis of proinsulin led to an increased proinsulin misfolding, which resulted in impairments of ProIR processing and insulin signaling in β cells. These results reveal for the first time that misfolded proinsulin can interact with ProIR in the ER, impairing intracellular processing of ProIR and leading to defective insulin signaling that may contribute to β-cell failure in both MIDY and T2D.—Liu, S., Li, X., Yang, J., Zhu, R., Fan, Z., Xu, X., Feng, W., Cui, J., Sun, J., Liu, M. Misfolded proinsulin impairs processing of precursor of insulin receptor and insulin signaling in β cells.

Insulin is a major regulator of glucose homeostasis. In pancreatic β cells, insulin biosynthesis starts from its precursor, preproinsulin, in the cytosol. Newly synthesized preproinsulin is translocated into the endoplasmic reticulum (ER), where it is cleaved by signal peptidase at the luminal side of the ER membrane to generate proinsulin (1, 2). Proinsulin then undergoes rapid oxidative folding in the ER, forming 3 highly conserved disulfide bonds (B7-A7, B19-A20, and A6-A11). Well-folded proinsulin exits from the ER, trafficking through the Golgi to the late secretory pathway where it is processed by prohormone convertases (PC1/3 and PC2) and carboxypeptidase E into mature insulin (3, 4). It has been long believed that these processes are highly efficient and occur naturally in pancreatic β cells. However, accumulating evidence indicates that the proinsulin folding process in the ER is not as efficient as previously thought and that it is sensitive to the changes of the ER folding environment (5, 6). Under normal physiologic conditions, about 10–20% of newly synthesized proinsulin fails to achieve its native folding state (7–10), and the amount of misfolded proinsulin can be further increased under some stress conditions (10, 11). Over the past decade, more than 50 new insulin gene mutations have been reported to cause proinsulin misfolding and mutant *INS*-gene–induced diabetes of youth (MIDY) (8, 9, 12), highlighting important pathogenesis of proinsulin misfolding in the development and progression of diabetes. However, the underlying mechanisms of β-cell failure caused by misfolded mutant proinsulin are still incompletely understood. Recent studies show that misfolded proinsulin causes inefficient β-cell growth, suggesting that survival signaling is impaired in β cells expressing misfolded proinsulin (13, 14).

Insulin receptor (IR)-mediated insulin signaling plays an important role in β-cell survival and function (15–17). IR is a tetrameric membrane protein that is composed of 2 extracellular α subunits and 2 transmembrane β subunits linked by disulfide bonds (18, 19). The precursor of IR (ProIR) is synthesized in the ER, where it undergoes multiple post-translational modification, including oxidative folding, glycosylation, and dimerization in the ER. Once it has achieved its native folding state, ProIR is exported to the Golgi apparatus, where it is proteolytically processed into mature IR prior to insertion into the plasma membrane (20, 21). Impaired ProIR processing caused by IR gene mutations can lead to severe insulin resistance in the patients with Donohue syndrome (22, 23). However, its role in MIDY and type 2 diabetes (T2D) is unknown. Herein we report that, in Akita mice expressing a mutant proinsulin C(A7)Y that is identical to one of the MIDY mutants (24, 25), misfolded proinsulin could abnormally interact with ProIR in the ER, impairing its intracellular processing and decreasing insulin signaling in β cells. More importantly, we found that, in the β cells of db/db mice with severe insulin resistance that was due to a defect in leptin receptor (26, 27), there was a significantly increased proinsulin misfolding. These misfolded proinsulin molecules caused ProIR processing impairment that contributed to defective insulin signaling of the β cells in db/db mice. These results indicate that proinsulin misfolding is not only a molecular basis of MIDY but also an important contributor of β-cell failure in T2D. This study reveals that defective insulin signaling that is due to ProIR maturation impairment caused by misfolded proinsulin is a novel mechanism of β-cell failure in both MIDY and T2D.

## MATERIALS AND METHODS

### Reagents and antibodies

Thapsigargin, DTT, cycloheximide, complete proteinase inhibitor, histopaque 1077 and collagenase were purchased from MilliporeSigma (Burlington, MA, USA). Lipofectamine 2000 and 4–12% Bis-Tris Gradient Gel were from Thermo Fisher Scientific (Waltham, MA, USA). MG132 was purchased from MilliporeSigma. Recombinant human insulin and PMSF were from Solarbio Life Sciences (Beijing, China). Phosphatase inhibitor was from Beyotime Biotechnology (Beijing, China). Protein A-agarose, antibodies of IR β subunit (IR_β_) and phosphorylated (p)-protein kinase B (AKT) (Thr308) were from Santa Cruz Biotechnology (Dallas, TX, USA). Antibodies against p–eukaryotic initiation factor 2α (eIF2α), eIF2α, p-IR_β_ (Tyr1150/1151), p-AKT (Ser473) and AKT were from Cell Signaling Technology (Danvers, MA, USA). Rabbit polyclonal anti–protein disulfide isomerase (PDI) was from Thermo Fisher Scientific. Rabbit polyclonal anti-TGN38 was a gift from Dr. Yanzhuang Wang (University of Michigan, Ann Arbor, MI, USA). Mouse anti–glyceraldehyde 3-phosphate dehydrogenase and β-tubulin were purchased from Sungene Biotech (Tianjin, China). [^125^I] labeled proinsulin and guinea pig anti-insulin was from MilliporeSigma. Proinsulin antibody was from Novus Biologicals (Centennial, CO, USA). A mouse monoclonal antibody (C-A junction) that recognizes a human proinsulin C-peptide–A-chain junction was raised by Abmart (Shanghai, China). Goat anti-mouse IgG Alexa Fluor 555 and goat anti-rabbit IgG Alexa Fluor 488 were from Thermo Fisher Scientific. Goat anti-mouse IgG horseradish peroxidase (HRP), goat anti-rabbit IgG HRP, and goat anti-guinea pig IgG HRP were purchased from Jackson ImmunoReserach (West Grove, PA, USA). DAPI was from SouthernBiotech (Birmingham, AL, USA).

### Plasmids

cDNAs encoding myc-tagged human preproinsulin, mutation C(A7)Y (Akita proinsulin) and Delcys proinsulin, in which all 6 cystine residues were mutated to serine and were constructed and amplified as previously described (9). The plasmid encoding human IR isoform B was purchased from Addgene (plasmid 24049; Watertown, MA, USA). Plasmids encoding human IR isoform A and Y(818)C mutant IR isomer B were constructed by Tsingke Biologic Technology (Beijing, China).

### Animals, oral glucose tolerance test, and islet isolation

C57BL/KsJ-LepR db mice (male) and Akita mice (male) were housed at the Animal Care Facility of Tianjin General Hospital in a temperature-controlled room on a 12-h light/dark cycle with free access to chow and water. All experimental protocols were approved by the Animal Care and Use Committee of Tianjin Medical University for Animal Experimentation, and all experiments were conducted according to the Chinese Council on Animal Care guidelines. Fasting blood glucose, insulin, and body weight of db/db mice were measured at 12 wk. For the oral glucose tolerance test, after being unfed overnight (16 h), all mice were orally administered 20% glucose (2 g/kg body weight) for glucose tolerance. Blood was collected from tail at 0 (basal level), 30, 60, 120 min after glucose intake, and then blood glucose was determined by using a glucose analyzer, Accu-Chek Performa meter (Roche, Basel, Switzerland). For islet isolation, the mice were euthanized after 6 h without food. Pancreatic islets were isolated by collagenase digestion, followed by density gradient centrifugation with Histopaque-1077 (MilliporeSigma). Isolated islets were handpicked under a dissecting microscope and collected for following culture and analysis as previously described (10).

### Cell culture, transfection, and Western blotting

Human embryonic kidney (HEK)293 cells were cultured in DMEM supplemented with 10% fetal bovine serum. INS-1 cells were cultured in Roswell Park Memorial Institute 1640 medium with 10% fetal bovine serum, 0.1% (v/v) penicillin/streptomycin (Solarbio Life Sciences), and 5 μl/L 2-ME (MilliporeSigma). All cell lines were incubated at 37°C in a humidified 95% air and 5% CO_2_ atmosphere. For transient transfection, HEK293 cells were seeded in 12-well or 6-well plates for 24 h, and then were transfected with 0.15–2.5 μg plasmid DNA as indicated. After 48 h transfection, cells were lysed on ice for immunoblotting or immunoprecipitation. For Western blotting, cell lysates or mouse tissues were homogenized in ice-cold RIPA (25 mM Tris-HCl, 10 mM EDTA, 100 mM NaCl, 1% Triton X-100, 0.1% SDS, 0.2% sodium deoxycholate) supplemented with proteinase inhibitor and phosphatase inhibitor, then centrifuged at 13,000 *g* at 4°C for 10 min. Collected supernatants were boiled for 10 min. Proteins were resolved on 4–12% NuPage gradient gels (Thermo Fisher Scientific) and transferred to nitrocellulose membranes. After blocked in 5% milk at room temperature for 1 h, membranes were incubated with primary antibodies at 4°C overnight followed by appropriate secondary antibodies conjugated with HRP.

### Coimmunoprecipitation and immunofluorescence

Cell lysates or pancreatic islets in coimmunoprecipitation (co-IP) buffer (5 mM EDTA, 0.1 M NaCl, 25 mM Tris-HCl, pH 7.4, 0.1% Triton X-100, and proteinase inhibitor) were collected as above. Antibodies indicated in the text were added to lysates and incubated at 4°C for 2 h, followed by incubation with protein A-agarose beads at 4°C for an additional 1 h. The bound proteins in the precipitants were resuspended in sample buffer, boiled, and analyzed by immunoblotting (28). For immunostaining, paraffin-embedded pancreases were cut into 5-μm sections. After heat-based antigen retrieval in citrate buffer, the sections were incubated with anti-proinsulin, anti-PDI, anti-TGN38, and anti-IR (N-term) as indicated, followed by secondary antibodies conjugated to appropriate Alexa Fluor. Fluorescent images were visualized using Axio Imager M2 (Carl Zeiss, Oberkochen, Germany).

### Statistical analysis

All data were processed with Prism software (GraphPad Software, La Jolla, CA, USA) and were presented as means ± sem. An unpaired Student’s *t* test and 1-way ANOVA were used to determine significance among groups. A value of *P* < 0.05 was considered statistically significant.

## RESULTS

### Misfolded proinsulin impairs ProIR processing and insulin signaling in Akita mice

**Figure 1:**
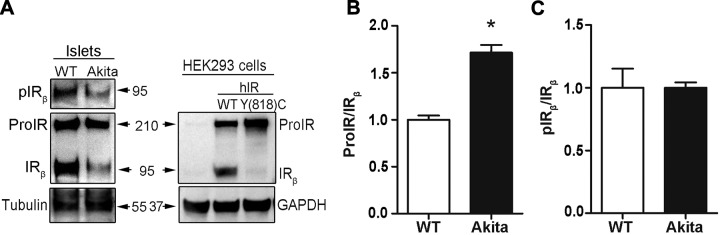
Impaired maturation of ProIR and insulin signaling in the islets of Akita mice. *A*) Freshly isolated islets from 6-wk-old Akita and WT mice were directly lysed. The total proteins were resolved in 10% SDS-PAGE, followed by Western blotting with anti-IR (reacts with both ProIR and IR) and anti–phosphor-IR_β_ as indicated (left panel). HEK293 cells were transfected with plasmids encoding human ProIR WT or processing defect mutant Y(818)C [hIR-WT or hIR-Y(818)C]. The cells were lysed after 48 h transfection. Unprocessed ProIR and mature IR were detected using anti-IR by Western blotting. *B*) Total ProIR and mature IR from the 3 independent experiments shown in *A* were quantified. The ratio of total ProIR and total IR_β_ was calculated, and ProIR/IR_β_ of WT islets was set as 1. Data were shown as the mean ± sem of 3 independent experiments. *P < 0.05. *C*) pIR_β_ and total IR_β_ from 3 independent experiments shown in *A* were quantified. The ratio of pIR_β_ and total IR_β_ was calculated, and pIR_β_/IR_β_ of WT islets was set as 1.

To unequivocally determine whether misfolded proinsulin can impair insulin signaling, we first examined the phosphorylation of IR in the isolated islets from 6-wk-old male Akita mice that carry a missense mutation in one of *Ins2* allele. We found that there is a significant decrease in pIR_β_ in Akita islets (**Fig. 1*A***, upper left panel). We then asked whether this impaired insulin signaling was caused by the defect of ProIR maturation. Two major forms, unprocessed ProIR and matured IR, are normally found in the islets. In order to distinguish the unprocessed ProIR from the mature IR, we expressed wild-type (WT) ProIR and a cleavage mutant ProIR Y(818)C in HEK293 cells. ProIR Y(818)C mutates at the ProIR proteolytic cleavage site and impairs its maturation (29, 30). Consistent with previous reports, the majority of ProIR Y(818)C indeed presented as unprocessed ProIR (Fig. 1*A*, right panel). Using these MW controls, we found that the mature IR (IR_β_) was slightly more abundant than the unprocessed ProIR in WT islets. In contrast, mature IR was markedly decreased, resulting in the ratio of ProIR/IR being significantly increased in Akita islets (Fig. 1*A, B*). Unlike ProIR/IR, the ratio of pIR_β_ and total IR_β_ (pIR_β_/IR_β_) was not different between WT and Akita islets (Fig. 1*A, C*), suggesting that the decreased IR-mediated insulin signaling in Akita islets was not due to the defect of IR phosphorylation but was caused by a decreased amount of mature IR that was due to a processing defect of ProIR (Fig. 1*A*–*C*). These data indicate that in Akita mice that express misfolded proinsulin, ProIR processing was impaired, which led to decreased IR phosphorylation and insulin signaling.

### Misfolded proinsulin fails to exit from the ER and impairs processing of ProIR into mature IR

Proinsulin is the most abundant protein in the ER of β cells, and proinsulin misfolding is the molecular basis of β-cell failure and diabetes in Akita mice (2, 12). We therefore examined the ER export of proinsulin by immunostaining pancreatic sections from Akita mice. In WT islets, proinsulin staining was most concentrated in the juxtanuclear region colocalized with a Golgi marker TGN38 (Supplemental Fig. S1*B*). However, in Akita islets, proinsulin became diffuse throughout the cells and colocalized with an ER marker PDI (Supplemental Fig. S1*A*), indicating that in Akita mice, misfolded proinsulin failed to export from the ER and advance to the Golgi. Interestingly, anti-IR immunostaining of Akita islets appeared to be different than that of the control islets, and more cells showed an abnormal anti-IR staining pattern that appeared to be accumulated/diffused inside the cells (Supplemental Fig. S1*C*, solid arrows). However, because there are no specific anti-IR and anti-ProIR available, these anti-IR immunostainings could not distinguish ProIR and IR in the cells. Nevertheless, anti-IR Western blotting clearly showed an increased unprocessed ProIR and an elevated ratio of ProIR/IR (Fig. 1), which suggests that misfolded proinsulin impairs ProIR processing in β cells, leading to decreased IR phosphorylation and insulin signaling.

**Figure 2:**
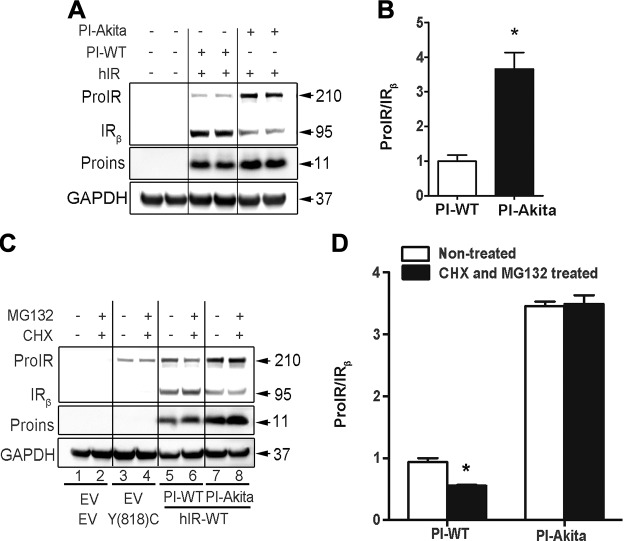
Misfolded proinsulin impairs processing of ProIR into mature IR. *A*) HEK293 cells were cotransfected with plasmids encoding human ProIR WT and human proinsulin WT or Akita (hIR + PI-WT or PI-Akita). The cells were lysed after 48 h transfection. Western blotting was performed to detect unprocessed ProIR, mature IR, and proinsulin. *B*) Total ProIR and mature IR from 3 independent experiments shown in *A* were quantified. The ratio of total ProIR and total IR_β_ was calculated, and ProIR/IR_β_ of hIR + PI-WT was set as 1. Data are expressed as the mean ± sem of 3 independent experiments. *P < 0.05. *C*) HEK293 cells were transfected with plasmids encoding human mutant ProIR Y(818)C, hIR WT and human proinsulin WT or Akita [hIR-Y(818)C + Empty vector, hIR + PI-WT or hIR + PI-Akita]. The cells were treated with cycloheximide (10 μg/ml) and MG132 (15 μM/ml) for 4 h after 36 h of transfection. The cells were then lysed and Western blotting was performed to detect unprocessed ProIR, mature IR, and proinsulin. *D*) Total ProIR and mature IR from 3 independent experiments shown in *C* were quantified. The ratio of total ProIR and total IR_β_ was calculated, and ProIR/IR_β_ of hIR + PI-WT without treatment was set as 1. Data are expressed as the mean ± sem of 3 independent experiments. *P < 0.05.

The Akita mice used in this study were around 6 wk of age and had already developed mild diabetes. In order to exclude the possibility that the impaired processing of ProIR in Akita islets was secondary to hyperglycemia, we coexpressed human ProIR with either proinsulin WT or proinsulin Akita in HEK293 cells. Again, expression of Akita proinsulin impaired the processing of ProIR, causing an increased ratio of ProIR/IR (**Fig. 2*A, B***), confirming that the effect of misfolded proinsulin on the processing of ProIR was independent of high blood glucose level. Furthermore, to directly examine ProIR processing in the presence or absence of misfolded Akita proinsulin, we treated cotransfected cells with cycloheximide (attenuated biosynthesis of newly made ProIR) and MG132 (a proteasomal inhibitor that prevents degradation of existing ProIR molecules) for 4 h. As shown in Fig. 2*C, D*, although ProIR mutant Y(818)C could not be processed because of mutation at cleavage site, ∼40% of WT ProIR underwent intracellular processing to form mature IR. However, in the presence of Akita proinsulin, WT ProIR failed to be processed into mature IR. Taken together, these data suggest that misfolded mutant proinsulin appears to be a direct cause accounting for impaired maturation of ProIR in Akita islets.

### ER stress impairs insulin signaling but does not affect ProIR processing

**Figure 3:**
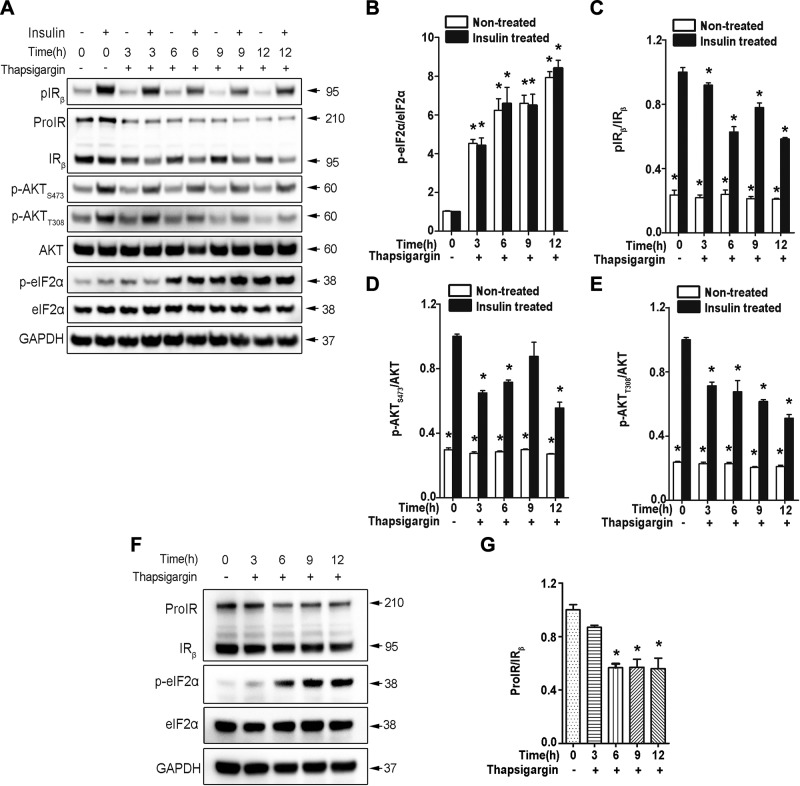
Misfolded proinsulin but not ER stress impairs processing of ProIR. *A*) INS-1 cells were incubated with thapsigargin (200 nM) for 0, 3, 6, 9, and 12 h as indicated. The cells were then stimulated with or without 100 nM insulin for 15 min. Western blotting was performed to evaluate p-eIF2α, eIF2α, unprocessed ProIR, mature IR, pIR_β_, p-AKT, and total AKT. *B*) p-eIF2α and total eIF2α from 3 independent experiments shown in *A* were quantified. The ratio of p-eIF2α and eIF2α was calculated, and p-eIF2α/eIF2α of the group treated with insulin alone was set as 1. *C*) pIR_β_ and total IR_β_ from 3 independent experiments shown in *A* were quantified. The ratio of pIR_β_ and total IR_β_ was calculated, and pIR_β_/IR_β_ of the group treated with insulin alone was set as 1. *D*) p-AKT at the site of serine473 (p-AKT_S473_) and total AKT from the 3 independent experiments shown in *A* were quantified. The ratio of p-AKT_S473_ and total AKT was calculated, and p-AKT_S473_/AKT of the group treated with insulin alone was set as 1. *E*) p-AKT at site of tyrosine308 (p-AKT_T308_) and total AKT from 3 independent experiments shown in *A* were quantified. The ratio of p-AKT_T308_ and total AKT was calculated, and p-AKT_T308_/AKT of the group treated with insulin alone was set as 1. Data are expressed as the mean ± sem of 3 independent experiments. *P < 0.05. *F*) INS-1 cells were incubated with thapsigargin (200 nM) for 0, 3, 6, 9, and 12 h as indicated. The cells were then were lysed and Western blotting was performed to evaluate p-eIF2α, eIF2α, unprocessed ProIR, mature IR, and glyceraldehyde 3-phosphate dehydrogenase. *G*) Total ProIR and mature IR from the 3 independent experiments without insulin treatment shown in *F* were quantified. The ratio of total ProIR and total IR_β_ was calculated, and ProIR/IR_β_ of the group without thapsigargin was set as 1. Data are expressed as the mean ± sem of 3 independent experiments. *P < 0.05.

There are at least 2 confounding factors that may affect processing of ProIR. First, proinsulin misfolding can induce ER stress (1), and ER stress is tightly associated with insulin resistance in insulin peripheral target tissues (31). It is necessary to determine whether ER stress affects ProIR maturation and insulin signaling in β cells. We therefore treated INS1 β-cell line for up to 12 h with thapsigargin, which depletes the ER calcium by inhibiting sarco/ER Ca2 + ATPase and inducing ER stress (32). Indeed, induction of ER stress could markedly blunt insulin-induced phosphorylation of IR and its downstream signaling AKT (**Fig. 3*A*–*E***). However, although the total amount of ProIR and IR were slightly decreased that may be secondary to a decrease of protein synthesis due to increased phosphorylation of eIF2a, ER stress did not adversely affect ProIR processing (Fig. *3F*). In fact, the ratio of ProIR/IR in thapsigargin-treated cells was even lower than that of the control cells (Fig. *3G*). It remains to be determined whether this decreased ratio of ProIR/IR is associated with an enhancement of ER protein export under the ER stress conditions (33, 34). Nevertheless, these data suggest that ER stress impairs insulin signaling by affecting IR phosphorylation and its downstream signaling. However, ER stress alone did not impair ProIR processing.

### Misfolded proinsulin specifically interacts with ProIR

**Figure 4:**
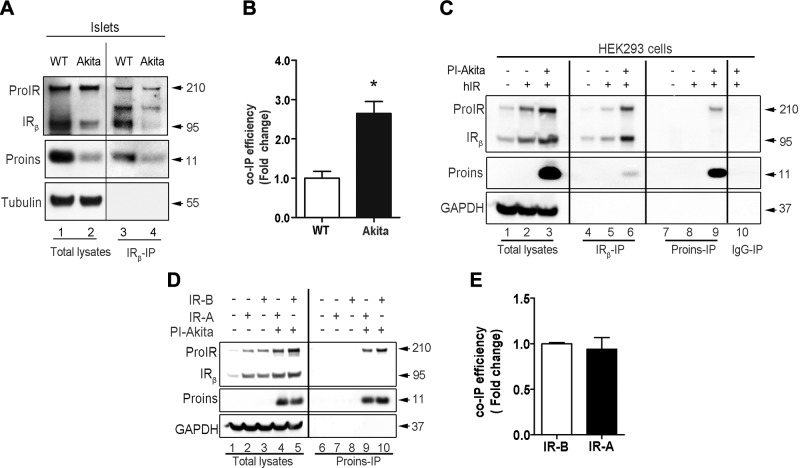
Proinsulin interacts with ProIR in the ER. *A*) Freshly isolated islets from 6-wk-old WT and Akita mice were directly lysed in co-IP lysis buffer followed by immunoprecipitation with anti-IR (reacts with both ProIR and IR). Proinsulin from total lysates (left panel) and from co-IP were detected using anti-proinsulin by Western blotting. *B*) Fold increases of relative amount of co-IP proinsulin WT and Akita. Quantification from at least 3 independent experiments as shown in *A*. Data are presented as means ± sem of 3 independent experiments. Significance values were determined by the Student’s *t* test. *P < 0.05. *C*) HEK293 cells were transfected with plasmids encoding human ProIR WT and human proinsulin Akita (hIR + PI-Akita). Co-IP analysis was performed using anti-proinsulin (does not react with mature insulin) or IR antibody (reacts with both ProIR and IR) after 48 h transfection. Akita proinsulin, ProIR, and IR from total lysates and co-IP were detected by Western blotting using anti-IR (upper panel) and anti-proinsulin (middle panel). Lane 10, a negative control of HEK293 cell lysates immunoprecipitated using the IgG. *D*) HEK293 cells were cotransfected with plasmids encoding human ProIR-A or ProIR-B with or without human proinsulin Akita (PI-Akita) as indicated. Co-IP analysis was performed using anti-proinsulin after 48 h of transfection. Akita proinsulin, ProIR, and IR from total lysates and co-IP were detected by Western blotting using anti-IR (upper panel) and anti-proinsulin (middle panel). *E*) Fold increases of relative amounts of co-IP ProIR. Quantification from at least 3 independent experiments shown in *D*. Data are presented as the mean ± sem of 3 independent experiments. Significance values were determined by the Student’s *t* test. *P < 0.05.

To further explore the underlying mechanism of misfolded proinsulin in IR maturation, we performed co-IP followed by Western blotting to determine whether proinsulin could interact with ProIR. In the isolated islets, anti-IR_β_ antibody could pull down proinsulin both from WT and Akita islets. However, the binding efficiency was higher in Akita islets (**Fig. 4*A, B***), and this co-IP experiment could not distinguish that the pulled down proinsulin was bound to IR or ProIR. We therefore coexpressed human ProIR isomer B and Akita proinsulin in HEK293 cells and did co-IP using antibodies against either IR or proinsulin. Again, we found that anti-IR antibody could pull down Akita proinsulin in cotransfected cells (Fig. 4*C*, lanes 4–6). More importantly, anti-proinsulin antibody did not pull down more abundant IR but specifically co-IP ProIR (Fig. 4*C*, lanes 7–9), indicating that proinsulin specifically interacts with ProIR. Because misfolded proinsulin is recognized by ER quality control system and is retained in the ER (1, 12), the interactions between misfolded proinsulin is very likely to occur in the ER.

It is worth noting that ProIR has 2 isomers, ProIR-A and ProIR-B. Although the 2 isoforms have only minimal differences in their affinity for insulin, it has been reported that IR-A have a higher affinity for proinsulin than do IR-B (35–37). It is unknown whether ProIR-A and ProIR-B interact with proinsulin differently. We therefore performed co-IP experiments in transfected HEK293 cells coexpressing Akita proinsulin with either ProIR-A or ProIR-B. Surprisingly, we found no significant difference in binding affinity of Akita proinsulin to the 2 isoforms ProIR-A and ProIR-B (Fig. 4*D, E*), suggesting that misfolded proinsulin may interact with ProIR *via* an unconventional way that does not involve conventional domains/residues mediating normal interactions between insulin and IR. We have previously shown that newly synthesized misfolded proinsulin molecules have free thiol, which can form disulfide-linked protein complexes in the ER (9, 25). We therefore hypothesized that misfolded proinsulin may interact with ProIR through abnormal intermolecular disulfide bonds. To test this hypothesis, we performed anti-proinsulin co-IP experiments in the cells coexpressing ProIR with either Akita proinsulin or Delcys proinsulin in which all 6 cystine residues were mutated to serine. We found that the detectable monomeric Akita proinsulin and co-IP ProIR were significantly lower in nonreducing condition compared with that in reducing conditions (Supplemental Fig. S2, lane 5 *vs.* 11). By contrast, in the cells coexpressing ProIR with proinsulin without cystine (Delcys), both monomeric Delcys and co-IP ProIR were detected at similar levels under both nonreducing and reducing conditions (Supplemental Fig. S2, lane 6 *vs.* 12), and interactions of ProIR with Delcys appear to be less than that with Akita (Supplemental Fig. S2, compared lanes 5 *vs.* 6). These data suggest that intermolecular disulfide bonds mediate at least partially the abnormal interactions between misfolded proinsulin and ProIR in the ER. Altogether, these results suggest that misfolded proinsulin specifically interacts with ProIR in the ER.

### Up-regulation of proinsulin synthesis and the ER overload lead to increased proinsulin misfolding

**Figure 5:**
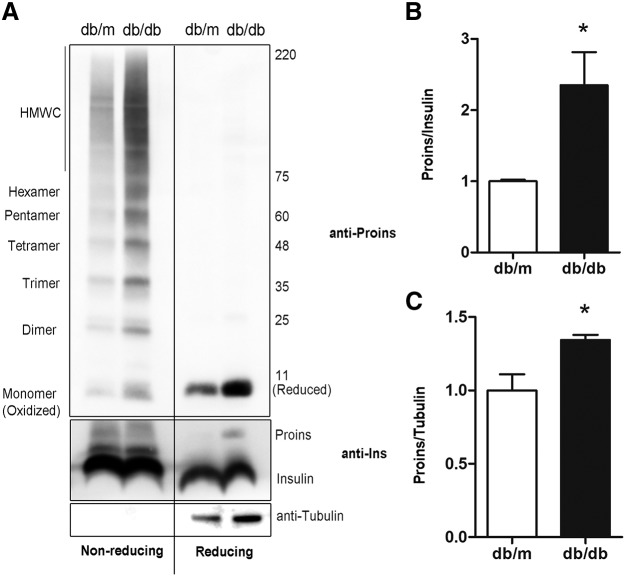
Misfolded proinsulin increases in the islets of db/db mice. *A*) Freshly isolated islets from 12-wk-old C57BL/KsJ-LepR db/db and lean control db/m mice were directly lysed. The total proteins were resolved in 4–12% gradient gel under nonreducing and reducing conditions, electrotransferred to nitrocellulose membrane followed by Western blotting with anti-proinsulin (upper panel) and anti-insulin (bottom panel) as indicated. *B*) Proinsulin and insulin from 3 independent experiments shown in *A* were quantified. The ratio of total proinsulin and insulin was calculated, and proinsulin/insulin of db/m islets was set as 1. Data were shown as the mean ± sem of 3 independent experiments. *P < 0.05. *C*) Proinsulin and tubulin from 3 independent experiments shown in *A* were quantified. The ratio of proinsulin and tubulin was calculated, and proinsulin/tubulin of db/m islets was set as 1.

We have previously reported that about 10–20% of newly synthesized proinsulin fails to achieve its native folding state (7, 10, 38). The amount of misfolded proinsulin can further increase when the regulation of protein synthesis is compromised because of an insufficiency/absence of eIF2⍺ and its kinase protein kinase R-like ER kinase (PERK) (10, 11). Herein, we asked whether proinsulin misfolding and defect in insulin signaling of β cells play a role in β-cell failure during the development and progression of T2D. We isolated islets from newly onset diabetes leptin-receptor–deficient db/db mice (Supplemental Fig. S3), in which proinsulin synthesis is significantlyup-regulated to compensate severe insulin resistance (39). Consistent with our previous findings (7, 38), in the lean db/m control islets, anti-proinsulin antibody (recognizing the junction between proinsulin C-peptide and A-chain) detected not only monomeric proinsulin but also disulfide-linked proinsulin complexes, including dimers, trimers, tetramers, pentamers, hexamers, and high MW complexes under nonreducing conditions (**Fig. 5*A***, upper panel, left). A measure of 100 mM reducing agent DTT broke disulfide bonds and made all these disulfide-linked complexes collapse into a single reduced monomeric proinsulin (Fig. 5*A*, bottom panel, right). Because correctly paired native disulfide bonds (B7-A7, B19-A20, and A6-A11) are required for correct folding of proinsulin (1, 9, 10), these intermolecular disulfide-linked proinsulin complexes are by definition misfolded. Importantly, in db/db islets, although total proinsulin level was markedly increased as expected, the misfolded proinsulin complexes were concurrently elevated (Fig. 5*A*, upper panel), and impaired for the ER export (Supplemental Fig. S4). The ratio of proinsulin to insulin was also elevated in db/db islets (Fig. 5*A*, bottom panel and Fig. 5*B*), implying that the intracellular processing of proinsulin is impaired in db/db mice. Collectively, these results indicated that misfolded proinsulin increased in the pancreatic islets of db/db mice.

### Impaired maturation of ProIR contributes to defective insulin signaling in the islets of db/db mice

**Figure 6:**
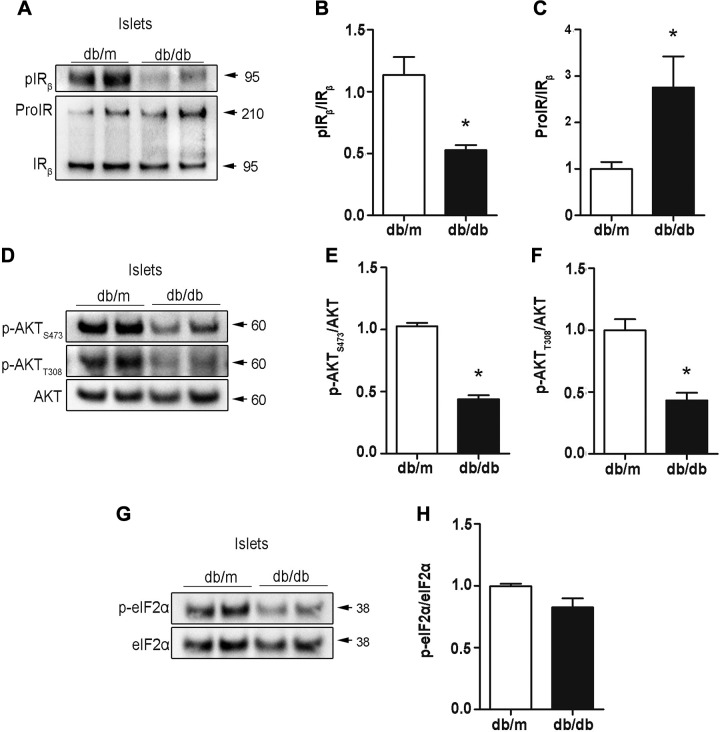
Impaired maturation of ProIR contributes to defective insulin signaling in the islets of db/db mice. Freshly isolated islets from 12-wk-old C57BL/KsJ-LepR db/db and lean control db/m mice were directly lysed. *A*) Western blotting was performed to detect unprocessed ProIR and mature IR using anti-IR and pIR_β_ using anti-pIR_β_. *B*) pIR_β_ and total IR_β_ from 3 independent experiments shown in *A* were quantified. The ratio of pIR_β_ and total IR_β_ was calculated, and pIR_β_/IR_β_ of db/m islets was set as 1. *C*) Total ProIR and mature IR from 3 independent experiments shown in *A* were quantified. The ratio of total ProIR and total IR_β_ was calculated, and ProIR/IR_β_ of db/m islets was set as 1. Data were shown as the mean ± sem of 3 independent experiments. *P < 0.05. *D*) Western blotting was performed to detect p-AKT at site of serine_473_ (p-AKT_s473_) or tyrosine_308_ (p-AKT_t308_) and total AKT. E. p-AKT_S473_ and total AKT from 3 independent experiments shown in *D* were quantified. The ratio of p-AKT_S473_ and total AKT was calculated, and p-AKT_S473_/AKT of db/m islets was set as 1. *F*) p-AKT_T308_ and total AKT from 3 independent experiments shown in *D* were quantified. The ratio of p-AKT_T308_ and total AKT was calculated, and p-AKT_T308_/AKT of db/m islets was set as 1. Data were shown as the mean ± sem of 3 independent experiments. *P < 0.05. *G*) Western blotting was performed to detect p-eIF2α and total eIF2α. *H*) p-eIF2α and total eIF2α from 3 independent experiments shown in *G* were quantified. The ratio of p-eIF2α and eIF2α was calculated, and p-eIF2α/ eIF2α of db/m islets was set as 1. Data were shown as the mean ± sem of 3 independent experiments. *P < 0.05.

Because we found increased misfolded proinsulin in db/db mice (Fig. 5), we asked whether maturation of ProIR was affected by misfolded proinsulin as we found in Akitamice (Figs. 1 and 2). Despite more than 6-fold increases of serum insulin level in db/db mice (Supplemental Fig. S3*C*), phospho-IR and its downstream PKB (AKT) were significantly decreased in islets of db/db. This appeared to be associated with impaired processing of ProIR as the ratio of ProIR/IR was elevated in db/db islets (**Fig. 6*A*–*F***). Interestingly, no increased phosphorylation of eIF2α was observed in db/db islets (Fig. 6*G, H*), indicating that there is no ER stress activation at least in the branch of PERK/eIF2α pathway. These data indicate that there is indeed insulin resistance in islets and that impaired ProIR maturation contributes to this defective insulin signaling in db/db mice.

## DISCUSSION

Insulin resistance in peripheral insulin target tissues is important in the pathogenesis of T2D. In this study, we found that defective insulin signaling also existed in pancreatic islets and may play a critical role in the development and progression of diabetes in both Akita and db/db mice. Mechanically, this defective insulin signaling resulted from defects in processing of ProIR, and the increased proinsulin misfolding in β cells appeared to be the underlying mechanism that contributed to impaired ProIR maturation. Specifically, we found that misfolded proinsulin increased not only in monogenic diabetes model Akita mice but also in insulin resistance model db/db mice. Misfolded proinsulin specially interacted with ProIR in the ER, impaired the intracellular processing of ProIR, and led to increased ratio of ProIR/IR and defective insulin signaling in islets of Akita and db/db mice. Insulin signaling plays an important role in β-cell function, survival, and compensation to peripheral insulin resistance (16, 17, 40, 41). The current study not only revealed a novel mechanism of β-cell failure but also shed light on a new potential therapeutic target to prevent or delay monogenic and T2D.

Both genetic and biologic evidence indicates that proinsulin misfolding is the molecular basis of MIDY (24, 25, 42, 43). Over the past decades, studies have revealed that misfolded proinsulin can cause β-cell failure and diabetes through 2 mechanisms. First, misfolded proinsulin fails to exit from the ER, causing ER stress and β-cell apoptosis (43–46). Second, we report that misfolded proinsulin can abnormally interact with coexpressed WT proinsulin in the ER, blocking the ER export of WT proinsulin, leading to insulin-deficient diabetes (8, 9, 25, 28, 38, 47, 48). Most recently, studies show that diabetes-causing misfolded proinsulin impairs β-cell expansion during development (13, 14, 46), suggesting that in addition to the 2 mechanisms mentioned above, impaired β-cell proliferation may also contribute to diabetes development. Therefore, in this study, we examined β-cell insulin signaling in Akita islets and found that phosphorylation of IR was indeed decreased, supporting the notion that defective insulin signaling in β cells expressed misfolded mutant proinsulin. Importantly, the decreased IR phosphorylation was mainly caused by impaired intracellular processing of ProIR, leading to decreased mature IR and increased ratio of ProIR/IR (Fig. 1 and Supplemental Fig. S1*C*). These data indicate that impaired ProIR processing and maturation are responsible for the defective insulin signaling in β cells expressing misfolded proinsulin.

How does misfolded proinsulin affect maturation of ProIR? In all eukaryotic cells, the ER serves as the primary folding compartment for secretory and membrane proteins. We and others have shown that misfolded proinsulin is recognized by the ER quality control system and retained in the ER (12, 38, 43, 44), suggesting that the effect of misfolded proinsulin on ProIR must occur in the ER. In addition, a growing body of evidence indicates that although misfolded proinsulin could block the ER export of coexpressed WT proinsulin, it did not affect glycosylation and secretion of coexpressed ⍺1-antitrypsin, suggesting that the generation function of the secretion pathway is preserved (8, 9, 38) and that the effect of misfolded proinsulin on ProIR must be specific. Competitive binding experiments show that proinsulin is able to bind to both IR and ProIR, despite low binding efficiency compared with that of insulin (49, 50). Given the high level of proinsulin in the ER of β cells, it is likely that proinsulin can interact with ProIR in the ER. We therefore hypothesize that when well-folded proinsulin binds to ProIR in the ER, both of them can exit from the ER and traffic forward to the Golgi and late secretory pathway. However, when misfolded proinsulin binds to ProIR in the ER, the anterograde trafficking of ProIR may be impaired because of the retention of misfolded proinsulin recognized by the ER quality control system. Indeed, co-IP experiments confirmed that both proinsulin WT and mutant could interact with ProIR (Fig. 4*A*). However, in the current study it is unknown whether proinsulin WT and mutant use the same way to bind the same site of ProIR. Given the fact that more percent of Akita proinsulin appears to bind to ProIR than that of WT proinsulin (Fig. 4*B*), it is unlikely that Akita proinsulin binds to ProIR in the same way as WT proinsulin does. In fact, our data suggest that abnormal intermolecular disulfide bonds may be involved in abnormal interactions between misfolded proinsulin and ProIR in the ER.

Proinsulin is the most predominant secretory protein loaded in the ER of β cells. Under high glucose, synthesis of proinsulin alone can account for 30–50% of total protein synthesis in the β cells (2, 3). Nevertheless, like some other proteins, the efficiency of proinsulin folding is less than perfect so that β cells continuously produce a certain fraction of misfolded proinsulin (2, 3, 10, 51). PERK/eIF2α plays a key role in initiation of proinsulin translation (52, 53). The pathophysiological significance of PERK/eIF2⍺ regulation is highlighted by Wolcott-Rallison syndrome, a homozygous PERK deficiency causing early onset diabetes (54). The diabetic phenotype in PERK null mice recapitulates the human disease (55). Importantly, in PERK/eIF2⍺-deficient/inefficient mice, more misfolded proinsulin is observed (10, 11), suggesting that dysregulation of proinsulin synthesis may overwhelm the ER folding capacity and cause proinsulin misfolding. Proinsulin oversynthesis is commonly seen at the time of onset of T2D in obese patients with insulin resistance (56). In this study, we examined proinsulin folding in db/db mice with severe insulin resistance and oversynthesis of proinsulin. We found that there was significantly increased misfolded disulfide-linked proinsulin complexes along with an increased ratio of proinsulin to insulin in db/db mice (Fig. 5), supporting the notions that proinsulin is predisposed to misfold in unfavorable ER folding environment and that increased misfolded proinsulin may contribute to β-cell failure in db/db mice.

How does increased misfolded proinsulin link to β-cell failure in db/db mice? Because we found that misfolded proinsulin could interact with ProIR in the ER and impair ProIR processing in Akita mice, we therefore asked whether increased proinsulin misfolding in db/db islets impairs ProIR processing. We examined the processing of ProIR and found that the ratio of ProIR/IR was increased, suggesting that the maturation of ProIR was impaired in db/db islets. Importantly, this impairment appeared to result in defective insulin signaling because we found that, despite being exposed to a much higher level of insulin, phosphorylation of IR and AKT of freshly isolated islets from db/db mice was significantly decreased (Fig. 6). These data suggest insulin resistance, which is commonly referred to as defective insulin signaling in the classic insulin target tissues, also exists in the islets. Because insulin signaling plays an important role in β-cell proliferation responding to peripheral insulin resistance (17, 40), the finding of β-cell insulin resistance reveals a novel mechanism of β-cell decompensation in T2D.

## Supplementary Material

This article includes supplemental data. Please visit *http://www.fasebj.org* to obtain this information.

## Abbreviations

AKT: protein kinase B
co-IP: coimmunoprecipitation
eIF2α: eukaryotic initiation factor 2α
ER: endoplasmic reticulum
HEK: human embryonic kidney
HRP: horseradish peroxidase
IR: insulin receptor
IR_β_: IR β subunit
MIDY: mutant *INS*-gene–induced diabetes of youth
PDI: protein disulfide isomerase
PERK: protein kinase R-like ER kinase
pIR_β_: phosphorylated IR_β_
ProIR: precursor of IR
T2D: type 2 diabetes
WT: wild type

## References

[1] Liu M., Weiss M. A., Arunagiri A., Yong J., Rege N., Sun J., Haataja L., Kaufman R. J., Arvan P. (2018) Biosynthesis, structure, and folding of the insulin precursor protein. Diabetes Obes. Metab. 20 (Suppl 2), 28–50 30230185 10.1111/dom.13378 PMC6463291

[2] Sun J., Cui J., He Q., Chen Z., Arvan P., Liu M. (2015) Proinsulin misfolding and endoplasmic reticulum stress during the development and progression of diabetes. Mol. Aspects Med. 42, 105–118 25579745 10.1016/j.mam.2015.01.001 PMC4404191

[3] Liu M., Wright J., Guo H., Xiong Y., Arvan P. (2014) Proinsulin entry and transit through the endoplasmic reticulum in pancreatic beta cells. Vitam. Horm. 95, 35–62 24559913 10.1016/B978-0-12-800174-5.00002-8

[4] Dodson G., Steiner D. (1998) The role of assembly in insulin’s biosynthesis. Curr. Opin. Struct. Biol. 8, 189–194 9631292 10.1016/s0959-440x(98)80037-7

[5] Zito E., Chin K. T., Blais J., Harding H. P., Ron D. (2010) ERO1-beta, a pancreas-specific disulfide oxidase, promotes insulin biogenesis and glucose homeostasis. J. Cell Biol. 188, 821–832; erratum: 189, 769 20308425 10.1083/jcb.200911086 PMC2845084

[6] Tsuchiya Y., Saito M., Kadokura H., Miyazaki J. I., Tashiro F., Imagawa Y., Iwawaki T., Kohno K. (2018) IRE1-XBP1 pathway regulates oxidative proinsulin folding in pancreatic β cells. J. Cell Biol. 217, 1287–1301; erratum: 218, 1764 29507125 10.1083/jcb.201707143 PMC5881499

[7] Guo H., Xiong Y., Witkowski P., Cui J., Wang L. J., Sun J., Lara-Lemus R., Haataja L., Hutchison K., Shan S. O., Arvan P., Liu M. (2014) Inefficient translocation of preproinsulin contributes to pancreatic β cell failure and late-onset diabetes. J. Biol. Chem. 289, 16290–16302 24770419 10.1074/jbc.M114.562355 PMC4047398

[8] Liu M., Hodish I., Haataja L., Lara-Lemus R., Rajpal G., Wright J., Arvan P. (2010) Proinsulin misfolding and diabetes: mutant INS gene-induced diabetes of youth. Trends Endocrinol. Metab. 21, 652–659 20724178 10.1016/j.tem.2010.07.001 PMC2967602

[9] Liu M., Haataja L., Wright J., Wickramasinghe N. P., Hua Q. X., Phillips N. F., Barbetti F., Weiss M. A., Arvan P. (2010) Mutant INS-gene induced diabetes of youth: proinsulin cysteine residues impose dominant-negative inhibition on wild-type proinsulin transport. PLoS One 5, e13333 10.1371/journal.pone.0013333 PMC2952628 20948967

[10] Liu M., Li Y., Cavener D., Arvan P. (2005) Proinsulin disulfide maturation and misfolding in the endoplasmic reticulum. J. Biol. Chem. 280, 13209–13212 15705595 10.1074/jbc.C400475200 PMC2527538

[11] Scheuner D., Vander Mierde D., Song B., Flamez D., Creemers J. W., Tsukamoto K., Ribick M., Schuit F. C., Kaufman R. J. (2005) Control of mRNA translation preserves endoplasmic reticulum function in beta cells and maintains glucose homeostasis. Nat. Med. 11, 757–764 15980866 10.1038/nm1259

[12] Liu M., Sun J., Cui J., Chen W., Guo H., Barbetti F., Arvan P. (2015) INS-gene mutations: from genetics and beta cell biology to clinical disease. Mol. Aspects Med. 42, 3–18 25542748 10.1016/j.mam.2014.12.001 PMC4404187

[13] Riahi Y., Israeli T., Yeroslaviz R., Chimenez S., Avrahami D., Stolovich-Rain M., Alter I., Sebag M., Polin N., Bernal-Mizrachi E., Dor Y., Cerasi E., Leibowitz G. (2018) Inhibition of mTORC1 by ER stress impairs neonatal β-cell expansion and predisposes to diabetes in the *Akita* mouse. eLife 7, e38472 10.7554/eLife.38472 PMC6294551 30412050

[14] Modi H., Johnson J. D. (2018) Folding mutations suppress early beta-cell proliferation. eLife 7, e43475 10.7554/eLife.43475 PMC6294546 30547883

[15] Kulkarni R. N., Brüning J. C., Winnay J. N., Postic C., Magnuson M. A., Kahn C. R. (1999) Tissue-specific knockout of the insulin receptor in pancreatic beta cells creates an insulin secretory defect similar to that in type 2 diabetes. Cell 96, 329–339 10025399 10.1016/s0092-8674(00)80546-2

[16] Liew C. W., Assmann A., Templin A. T., Raum J. C., Lipson K. L., Rajan S., Qiang G., Hu J., Kawamori D., Lindberg I., Philipson L. H., Sonenberg N., Goldfine A. B., Stoffers D. A., Mirmira R. G., Urano F., Kulkarni R. N. (2014) Insulin regulates carboxypeptidase E by modulating translation initiation scaffolding protein eIF4G1 in pancreatic β cells. Proc. Natl. Acad. Sci. USA 111, E2319–E2328 24843127 10.1073/pnas.1323066111 PMC4050564

[17] Wang Q., Jin T. (2009) The role of insulin signaling in the development of β-cell dysfunction and diabetes. Islets 1, 95–101 21099255 10.4161/isl.1.2.9263

[18] Hubbard S. R. (2013) The insulin receptor: both a prototypical and atypical receptor tyrosine kinase. Cold Spring Harb. Perspect. Biol. 5, a008946 10.1101/cshperspect.a008946 PMC3578362 23457259

[19] Sparrow L. G., McKern N. M., Gorman J. J., Strike P. M., Robinson C. P., Bentley J. D., Ward C. W. (1997) The disulfide bonds in the C-terminal domains of the human insulin receptor ectodomain. J. Biol. Chem. 272, 29460–29467 9368005 10.1074/jbc.272.47.29460

[20] Lane M. D., Ronnett G., Slieker L. J., Kohanski R. A., Olson T. L. (1985) Post-translational processing and activation of insulin and EGF proreceptors. Biochimie 67, 1069–1080 3000457 10.1016/s0300-9084(85)80104-8

[21] Ronnett G. V., Knutson V. P., Kohanski R. A., Simpson T. L., Lane M. D. (1984) Role of glycosylation in the processing of newly translated insulin proreceptor in 3T3-L1 adipocytes. J. Biol. Chem. 259, 4566–4575 6368559

[22] Falik Zaccai T. C., Kalfon L., Klar A., Elisha M. B., Hurvitz H., Weingarten G., Chechik E., Fleisher Sheffer V., Haj Yahya R., Meidan G., Gross-Kieselstein E., Bauman D., Hershkovitz S., Yaron Y., Orr-Urtreger A., Wertheimer E. (2014) Two novel mutations identified in familial cases with Donohue syndrome. Mol. Genet. Genomic Med. 2, 64–72 24498630 10.1002/mgg3.43 PMC3907912

[23] Stenson P. D., Mort M., Ball E. V., Howells K., Phillips A. D., Thomas N. S. T., Cooper D. N. (2009) The human gene mutation database: 2008 update. Genome Med. 1, 13 19348700 10.1186/gm13 PMC2651586

[24] Støy J., Edghill E. L., Flanagan S. E., Ye H., Paz V. P., Pluzhnikov A., Below J. E., Hayes M. G., Cox N. J., Lipkind G. M., Lipton R. B., Greeley S. A. W., Patch A.-M., Ellard S., Steiner D. F., Hattersley A. T., Philipson L. H., Bell G. I.; Neonatal Diabetes International Collaborative Group (2007) Insulin gene mutations as a cause of permanent neonatal diabetes. Proc. Natl. Acad. Sci. USA 104, 15040–15044 17855560 10.1073/pnas.0707291104 PMC1986609

[25] Liu M., Hodish I., Rhodes C. J., Arvan P. (2007) Proinsulin maturation, misfolding, and proteotoxicity. Proc. Natl. Acad. Sci. USA 104, 15841–15846 17898179 10.1073/pnas.0702697104 PMC2000385

[26] Chua S. C. Jr., Chung W. K., Wu-Peng X. S., Zhang Y., Liu S.-M., Tartaglia L., Leibel R. L. (1996) Phenotypes of mouse diabetes and rat fatty due to mutations in the OB (leptin) receptor. Science 271, 994–996 8584938 10.1126/science.271.5251.994

[27] Kodama H., Fujita M., Yamaguchi I. (1994) Development of hyperglycaemia and insulin resistance in conscious genetically diabetic (C57BL/KsJ-db/db) mice. Diabetologia 37, 739–744 7988774 10.1007/BF00404329

[28] Haataja L., Snapp E., Wright J., Liu M., Hardy A. B., Wheeler M. B., Markwardt M. L., Rizzo M., Arvan P. (2013) Proinsulin intermolecular interactions during secretory trafficking in pancreatic β cells. J. Biol. Chem. 288, 1896–1906 23223446 10.1074/jbc.M112.420018 PMC3548498

[29] Van der Vorm E. R., Kuipers A., Kielkopf-Renner S., Krans H. M., Möller W., Maassen J. A. (1994) A mutation in the insulin receptor that impairs proreceptor processing but not insulin binding. J. Biol. Chem. 269, 14297–14302 8188715

[30] Nobile S., Semple R. K., Carnielli V. P. (2012) A novel mutation of the insulin receptor gene in a preterm infant with Donohue syndrome and heart failure. J. Pediatr. Endocrinol. Metab. 25, 363–366 22768670 10.1515/jpem-2011-0448

[31] Ozcan U., Yilmaz E., Ozcan L., Furuhashi M., Vaillancourt E., Smith R. O., Görgün C. Z., Hotamisligil G. S. (2006) Chemical chaperones reduce ER stress and restore glucose homeostasis in a mouse model of type 2 diabetes. Science 313, 1137–1140 16931765 10.1126/science.1128294 PMC4741373

[32] Richter M., Vidovic N., Honrath B., Mahavadi P., Dodel R., Dolga A. M., Culmsee C. (2016) Activation of SK2 channels preserves ER Ca^2+^ homeostasis and protects against ER stress-induced cell death. Cell Death Differ. 23, 814–827 26586570 10.1038/cdd.2015.146 PMC4832102

[33] Zhu R., Li X., Xu J., Barrabi C., Kekulandara D., Woods J., Chen X., Liu M. (2019) Defective endoplasmic reticulum export causes proinsulin misfolding in pancreatic β cells. Mol. Cell. Endocrinol. 493, 110470 10.1016/j.mce.2019.110470 PMC6613978 31158417

[34] Shaheen A. (2018) Effect of the unfolded protein response on ER protein export: a potential new mechanism to relieve ER stress. Cell Stress Chaperones 23, 797–806 29730847 10.1007/s12192-018-0905-2 PMC6111102

[35] Malaguarnera R., Sacco A., Voci C., Pandini G., Vigneri R., Belfiore A. (2012) Proinsulin binds with high affinity the insulin receptor isoform A and predominantly activates the mitogenic pathway. Endocrinology 153, 2152–2163 22355074 10.1210/en.2011-1843

[36] De Meyts P. (2012) The insulin receptor isoform A: a mitogenic proinsulin receptor? Endocrinology 153, 2054–2056 22523330 10.1210/en.2012-1234

[37] Bass J., Chiu G., Argon Y., Steiner D. F. (1998) Folding of insulin receptor monomers is facilitated by the molecular chaperones calnexin and calreticulin and impaired by rapid dimerization. J. Cell Biol. 141, 637–646 9566965 10.1083/jcb.141.3.637 PMC2132748

[38] Liu M., Lara-Lemus R., Shan S. O., Wright J., Haataja L., Barbetti F., Guo H., Larkin D., Arvan P. (2012) Impaired cleavage of preproinsulin signal peptide linked to autosomal-dominant diabetes. Diabetes 61, 828–837 22357960 10.2337/db11-0878 PMC3314357

[39] Alarcon C., Boland B. B., Uchizono Y., Moore P. C., Peterson B., Rajan S., Rhodes O. S., Noske A. B., Haataja L., Arvan P., Marsh B. J., Austin J., Rhodes C. J. (2016) Pancreatic β-cell adaptive plasticity in obesity increases insulin production but adversely affects secretory function. Diabetes 65, 438–450 26307586 10.2337/db15-0792 PMC4747460

[40] Okada T., Liew C. W., Hu J., Hinault C., Michael M. D., Krtzfeldt J., Yin C., Holzenberger M., Stoffel M., Kulkarni R. N. (2007) Insulin receptors in beta-cells are critical for islet compensatory growth response to insulin resistance. Proc. Natl. Acad. Sci. USA 104, 8977–8982 17416680 10.1073/pnas.0608703104 PMC1885613

[41] Kitamoto T., Sakurai K., Lee E. Y., Yokote K., Accili D., Miki T. (2018) Distinct roles of systemic and local actions of insulin on pancreatic β-cells. Metabolism 82, 100–110 29320716 10.1016/j.metabol.2017.12.017 PMC7391221

[42] Edghill E. L., Flanagan S. E., Patch A.-M., Boustred C., Parrish A., Shields B., Shepherd M. H., Hussain K., Kapoor R. R., Malecki M., MacDonald M. J., Støy J., Steiner D. F., Philipson L. H., Bell G. I., Hattersley A. T., Ellard S.; Neonatal Diabetes International Collaborative Group (2008) Insulin mutation screening in 1,044 patients with diabetes: mutations in the INS gene are a common cause of neonatal diabetes but a rare cause of diabetes diagnosed in childhood or adulthood. Diabetes 57, 1034–1042 18162506 10.2337/db07-1405 PMC7611804

[43] Colombo C., Porzio O., Liu M., Massa O., Vasta M., Salardi S., Beccaria L., Monciotti C., Toni S., Pedersen O., Hansen T., Federici L., Pesavento R., Cadario F., Federici G., Ghirri P., Arvan P., Iafusco D., Barbetti F.; Early Onset Diabetes Study Group of the Italian Society of Pediatric Endocrinology and Diabetes (SIEDP) (2008) Seven mutations in the human insulin gene linked to permanent neonatal/infancy-onset diabetes mellitus. J. Clin. Invest. 118, 2148–2156 18451997 10.1172/JCI33777 PMC2350430

[44] Park S.-Y., Ye H., Steiner D. F., Bell G. I. (2010) Mutant proinsulin proteins associated with neonatal diabetes are retained in the endoplasmic reticulum and not efficiently secreted. Biochem. Biophys. Res. Commun. 391, 1449–1454 20034470 10.1016/j.bbrc.2009.12.090 PMC2817945

[45] Izumi T., Yokota-Hashimoto H., Zhao S., Wang J., Halban P. A., Takeuchi T. (2003) Dominant negative pathogenesis by mutant proinsulin in the Akita diabetic mouse. Diabetes 52, 409–416 12540615 10.2337/diabetes.52.2.409

[46] Balboa D., Saarimäki-Vire J., Borshagovski D., Survila M., Lindholm P., Galli E., Eurola S., Ustinov J., Grym H., Huopio H., Partanen J., Wartiovaara K., Otonkoski T. (2018) Insulin mutations impair beta-cell development in a patient-derived iPSC model of neonatal diabetes. eLife 7, e38519 10.7554/eLife.38519 PMC6294552 30412052

[47] Hodish I., Liu M., Rajpal G., Larkin D., Holz R. W., Adams A., Liu L., Arvan P. (2010) Misfolded proinsulin affects bystander proinsulin in neonatal diabetes. J. Biol. Chem. 285, 685–694 19880509 10.1074/jbc.M109.038042 PMC2804216

[48] Wright J., Wang X., Haataja L., Kellogg A. P., Lee J., Liu M., Arvan P. (2013) Dominant protein interactions that influence the pathogenesis of conformational diseases. J. Clin. Invest. 123, 3124–3134 23722904 10.1172/JCI67260 PMC3696544

[49] Sugibayashi M., Shigeta Y., Teraoka H., Kobayashi M. (1992) Characterization of unprocessed insulin proreceptors in COS 7 cells transfected with cDNA with Arg735----Ser735 point mutation at the cleavage site. Metabolism 41, 820–826 1640858 10.1016/0026-0495(92)90161-3

[50] Lee H. C., Kim S.-J., Kim K.-S., Shin H.-C., Yoon J.-W. (2000) Remission in models of type 1 diabetes by gene therapy using a single-chain insulin analogue. Nature 408, 483–488 11100731 10.1038/35044106

[51] Schubert U., Antón L. C., Gibbs J., Norbury C. C., Yewdell J. W., Bennink J. R. (2000) Rapid degradation of a large fraction of newly synthesized proteins by proteasomes. Nature 404, 770–774 10783891 10.1038/35008096

[52] Lipson K. L., Fonseca S. G., Ishigaki S., Nguyen L. X., Foss E., Bortell R., Rossini A. A., Urano F. (2006) Regulation of insulin biosynthesis in pancreatic beta cells by an endoplasmic reticulum-resident protein kinase IRE1. Cell Metab. 4, 245–254 16950141 10.1016/j.cmet.2006.07.007

[53] Zhang P., McGrath B., Li S., Frank A., Zambito F., Reinert J., Gannon M., Ma K., McNaughton K., Cavener D. R. (2002) The PERK eukaryotic initiation factor 2 α kinase is required for the development of the skeletal system, postnatal growth, and the function and viability of the pancreas. Mol. Cell. Biol. 22, 3864–3874 11997520 10.1128/MCB.22.11.3864-3874.2002 PMC133833

[54] Castelnau P., Le Merrer M., Diatloff-Zito C., Marquis E., Tête M. J., Robert J. J. (2000) Wolcott-Rallison syndrome: a case with endocrine and exocrine pancreatic deficiency and pancreatic hypotrophy. Eur. J. Pediatr. 159, 631–633 10968248 10.1007/pl00008394

[55] Harding H. P., Zeng H., Zhang Y., Jungries R., Chung P., Plesken H., Sabatini D. D., Ron D. (2001) Diabetes mellitus and exocrine pancreatic dysfunction in perk-/- mice reveals a role for translational control in secretory cell survival. Mol. Cell 7, 1153–1163 11430819 10.1016/s1097-2765(01)00264-7

[56] Prentki M., Nolan C. J. (2006) Islet beta cell failure in type 2 diabetes. J. Clin. Invest. 116, 1802–1812 16823478 10.1172/JCI29103 PMC1483155

