# Supplementary figures and images for: Misfolded proinsulin impairs processing of precursor of insulin receptor and insulin signaling in β cells
Source: FASEB J. 2019 Aug 1;33(10):11338–48. doi: 10.1096/fj.201900442R (PMC6766638; doi:10.1096/fj.201900442R)

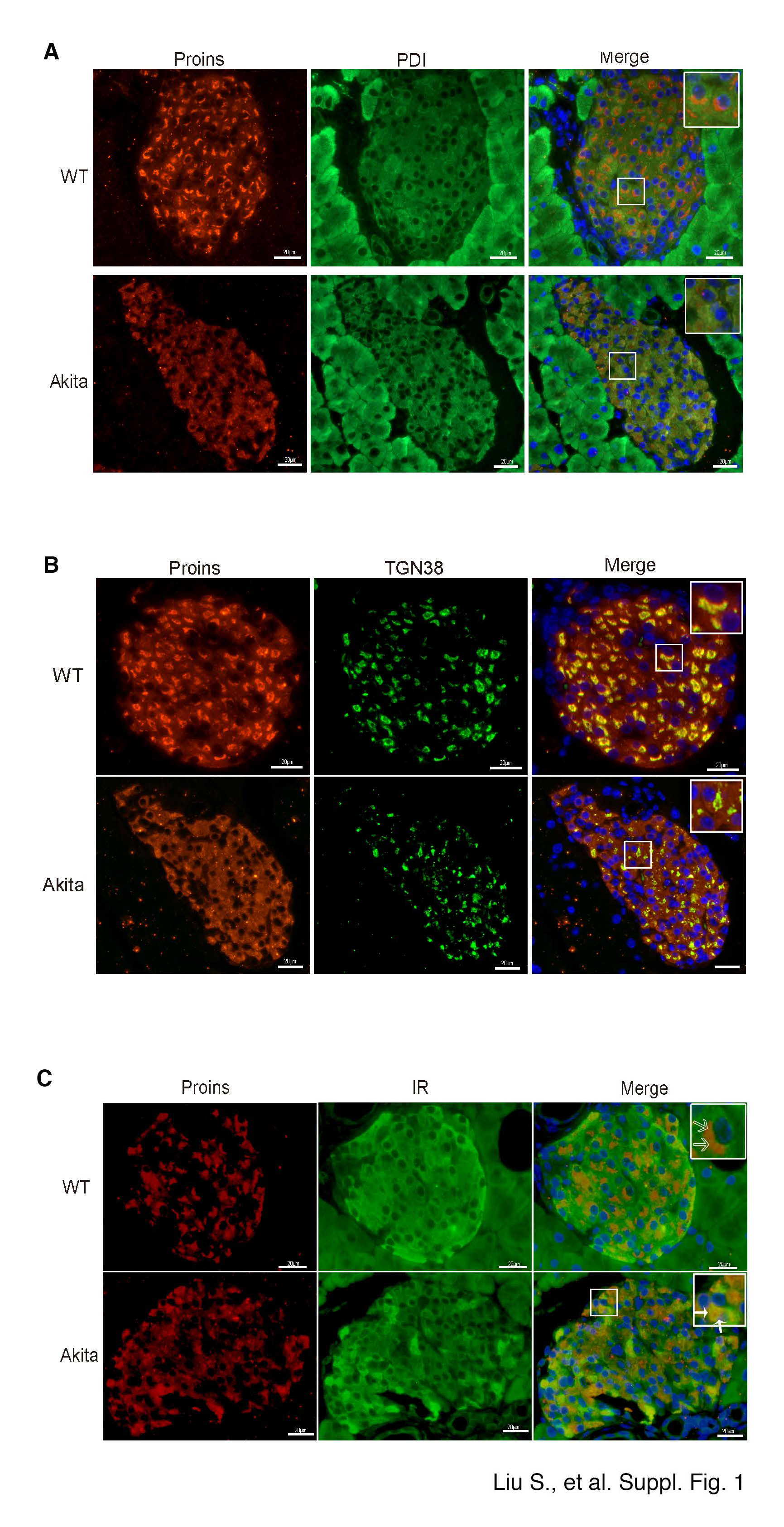

Supplement: Supplementary file 1 [file fj.201900442R.sf1.jpg]

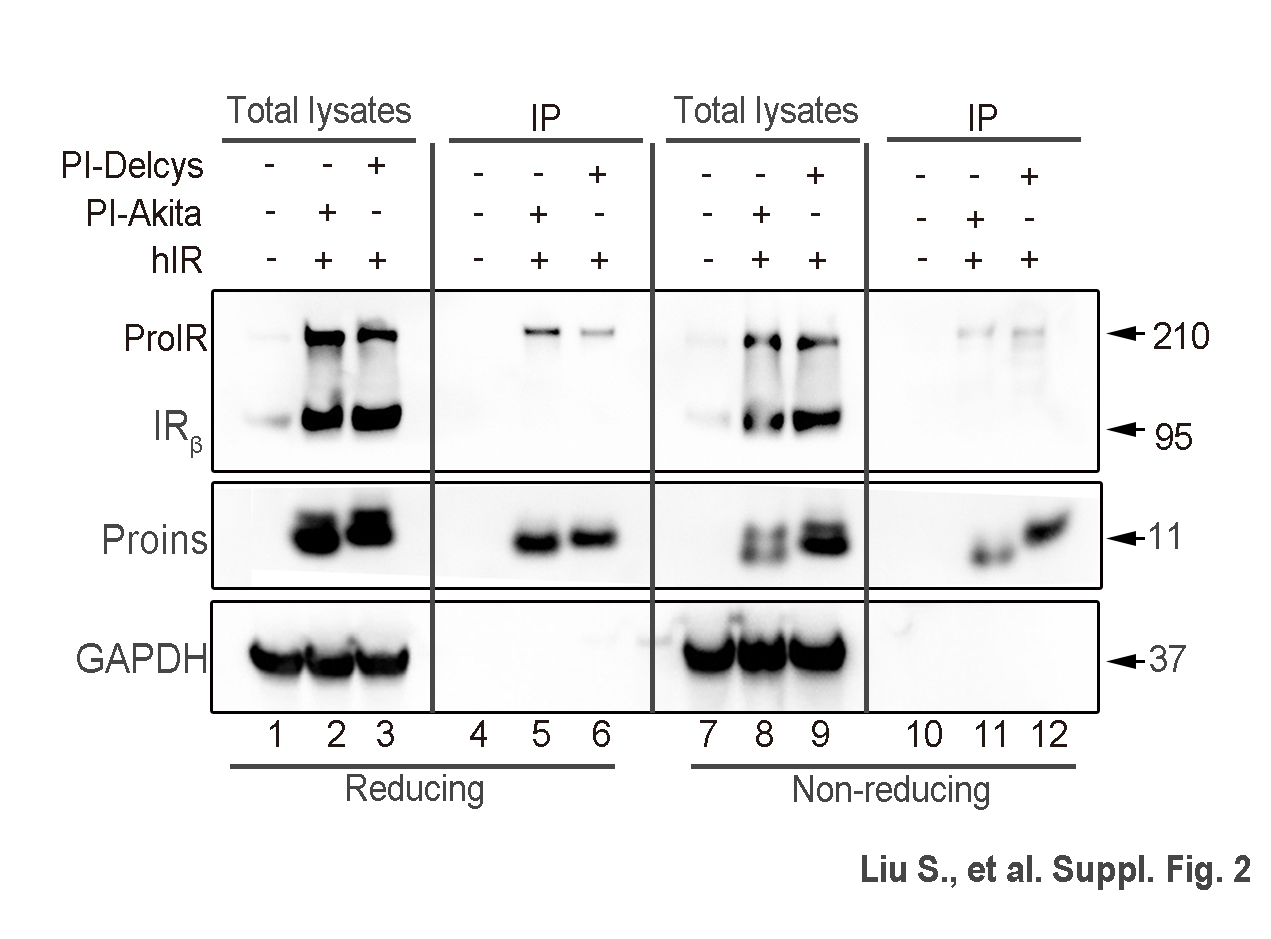

Supplement: Supplementary file 2 [file fj.201900442R.sf2.jpg]

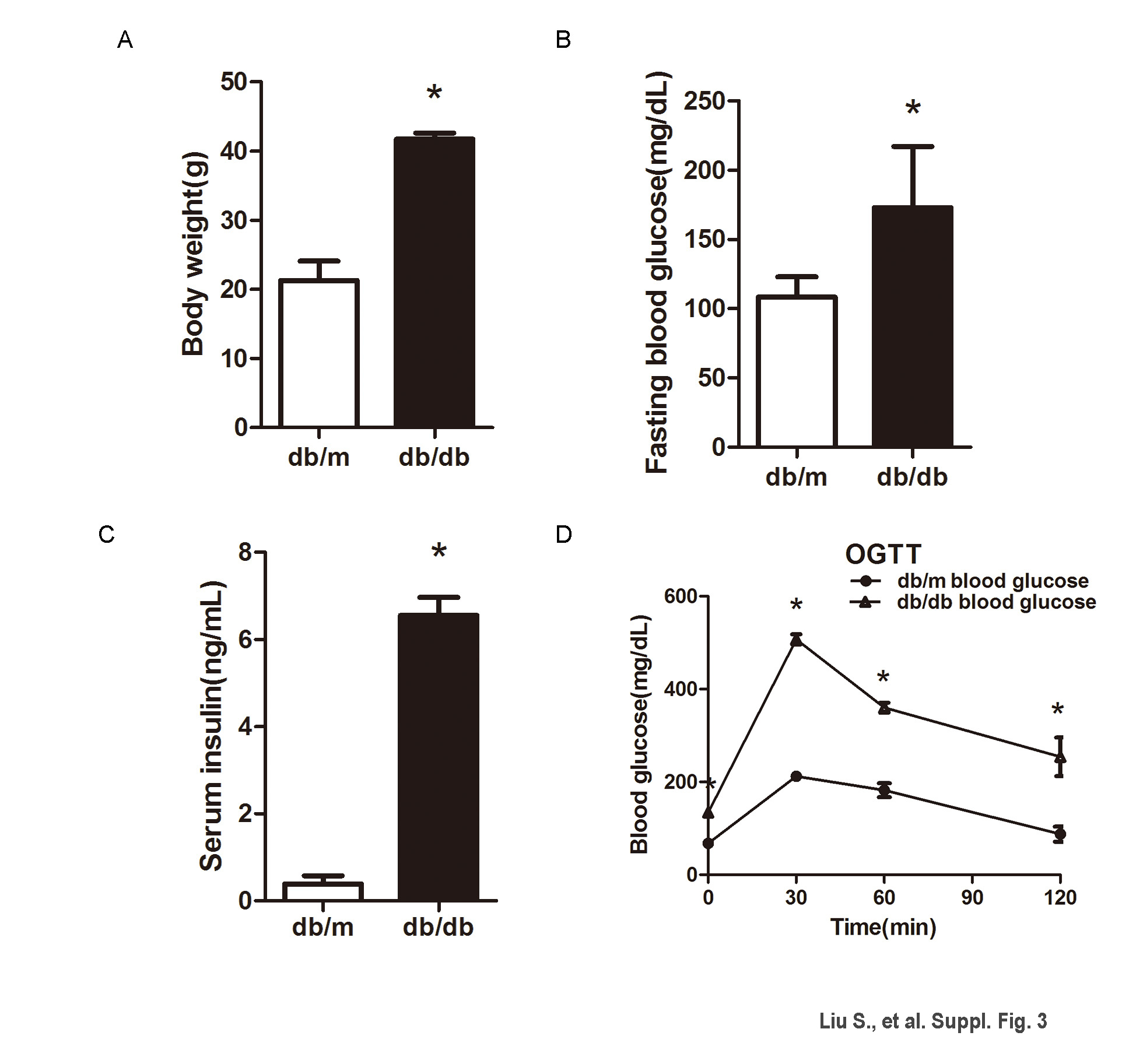

Supplement: Supplementary file 3 [file fj.201900442R.sf3.jpg]

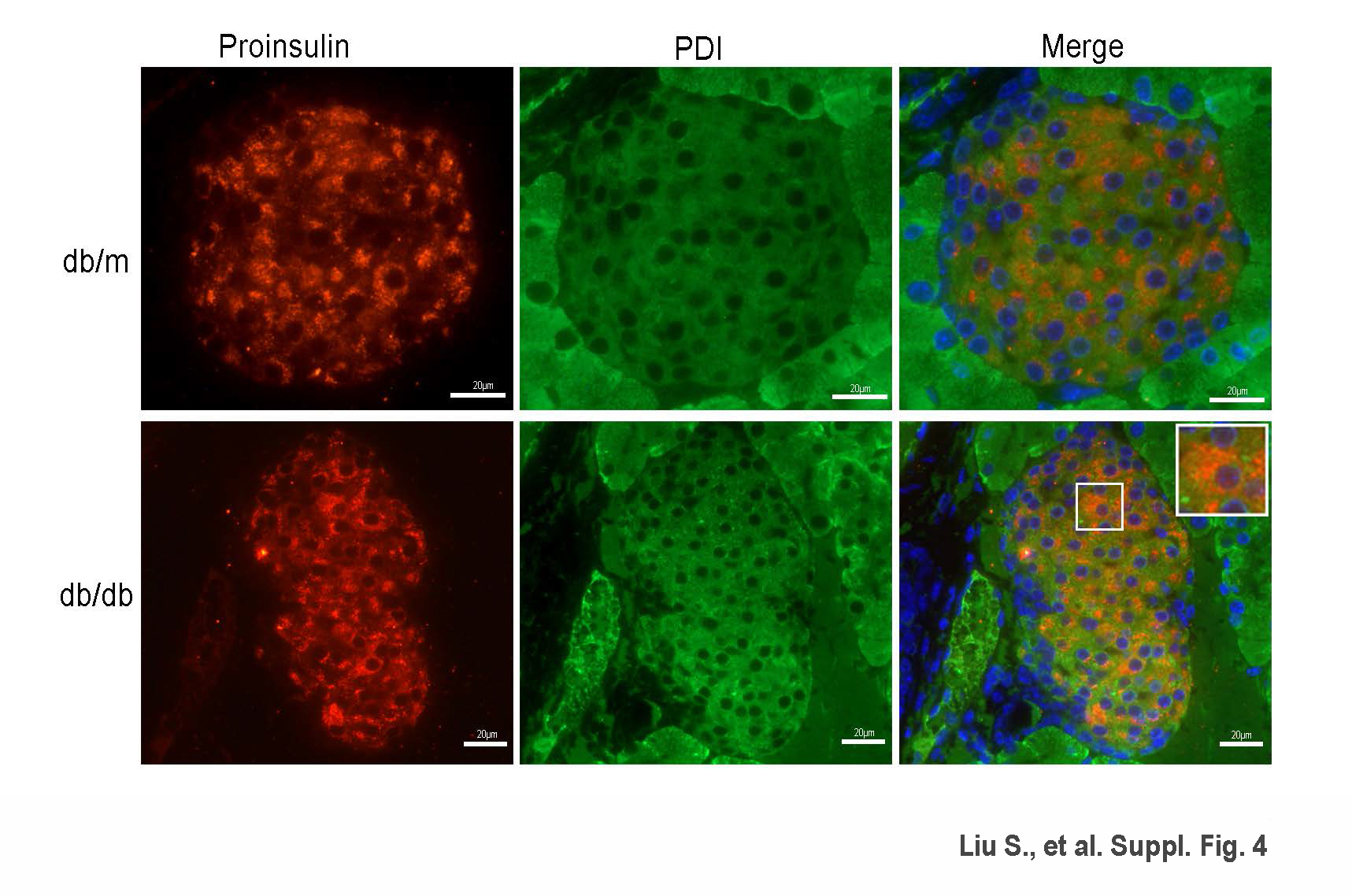

Supplement: Supplementary file 4 [file fj.201900442R.sf4.jpg]

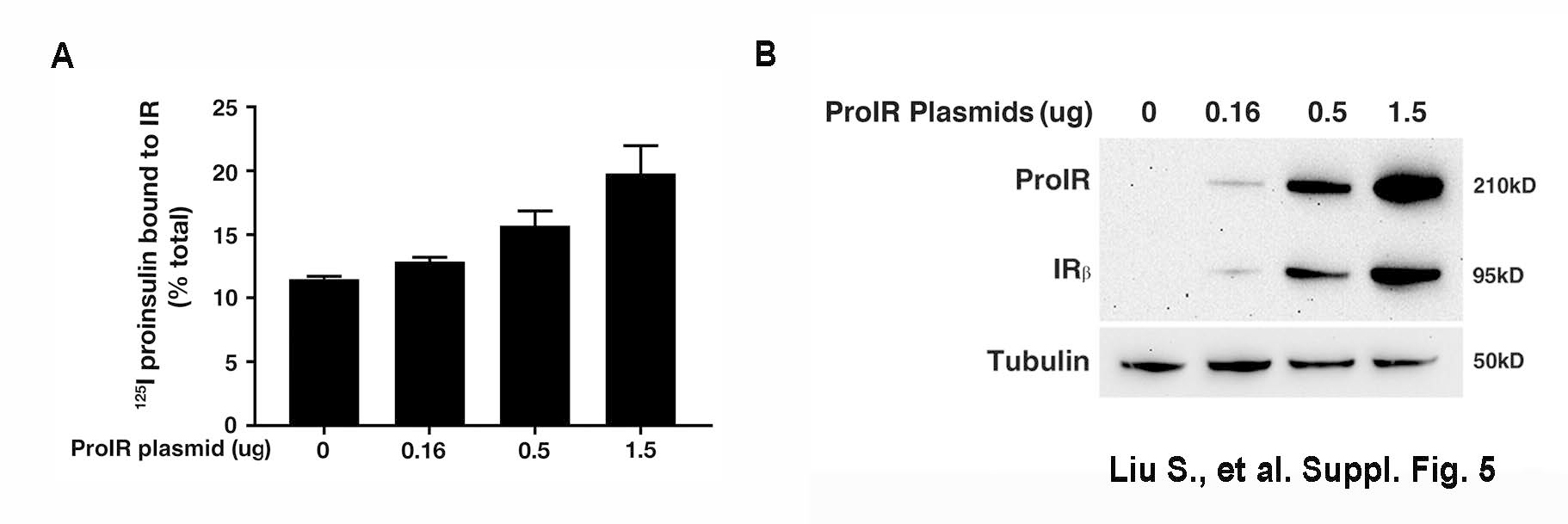

Supplement: Supplementary file 5 [file fj.201900442R.sf5.jpg]
