# Supplementary material for: Misfolded proinsulin impairs processing of precursor of insulin receptor and insulin signaling in β cells
Source: FASEB J. 2019 Aug 1;33(10):11338–48. doi: 10.1096/fj.201900442R (PMC6766638; doi:10.1096/fj.201900442R)
Supplement: Supplementary file 6 [file fj.201900442R.sd1.docx]

**Supplemental figure 1. Proinsulin fails to advance to the Golgi in the islets of Akita mice.**

**A**. Pancreatic sections of 6-week-old Akita and wild-type mice were double-immunostained with anti-proinsulin (red) and PDI (green). The nucleus was counterstained with DAPI (blue). **B**. Pancreatic sections of 6-week-old Akita and wild-type mice were double-immunostained with anti-proinsulin (red) and anti-TGN38 (green). The nucleus was counterstained with DAPI (blue). **C**. Pancreatic sections of 6-week-old Akita and wild-type mice were double-immunostained with anti-proinsulin (red) and anti-IR (green). The nucleus was counterstained with DAPI (blue).

**Supplemental figure 2. Abnormal intermolecular disulfide bonds contribute to abnormal interactions of misfolded proinsulin and ProIR in the ER.**

HEK293 cells were co-transfected with plasmids encoding ProIR with Akita proinsulin or proinsulin which harbors no cystine (Delcys). At 48 hours post-transfection, the cells were treated with MG132(15μM/mL) for 2 hours. The cells were lysed and co-IPed with anti-Proinsulin followed by analysis by western blotting under both non-reducing and reducing (with 100mM DTT) conditions.

**Supplemental figure 3. Body weight, fasting blood glucose, plasma insulin, and OGTT of db/db mice and db/m mice.**

**A**. Body weight, **B**. fasting blood glucose, **C**. serum insulin, and **D.** OGTT were performed using 12-week-old C57BL/KsJ-LepR db/db and lean control db/m mice. Data are expressed as mean ± SEM of three independent experiments. * stands for P < 0.05.

**Supplemental Figure 4. Proinsulin accumulates in the ER in the islets of db/db mice.**

Pancreatic sections of 12-week-old db/db mice and their littermate control were double-immunostained with anti-proinsulin (red) and PDI (green). The nucleus was counterstained with DAPI (blue).

**Supplemental Figure 5. Proinsulin dose-dependently binds to insulin receptor.**

**A.** HEK293 cells in 6 well plate were transfected with 0, 0.16, 0.5 or 1.5ug plasmid encoding human ProIR WT. At 24 hours post-transfection, the cells were split into 12 well plates, and cultured for additional 24 hours. The cells were then washed with cold PBS and incubated with ^125^I labeled proinsulin in PBS at 4℃ for 4 hours. The supernatants were collected, cells were harvested, and amounts of ^125^I labeled proinsulin in the supernatants and cells were countered by a gamma counter. The percentage of ^125^I labeled proinsulin bound to the cells were determined by calculating percentage of ^125^I labeled proinsulin in cell lysates verse total counts of supernatants and cell lysates. **B**. The cells came from parallel samples with Suppl. Fig 5A were lysed. The expression levels of ProIR and IR in the cells transfected with different amounts of ProIR plasmid were analyzed by western blotting using anti-IR.
